# Supplementary material for: Gait stability in ambulant children with cerebral palsy during dual tasks
Source: PLoS One. 2022 Jun 22;17(6):e0270145. doi: 10.1371/journal.pone.0270145 (PMC9216605; doi:10.1371/journal.pone.0270145)
Supplement: Attachment — Submitted filename: Responses-to-reviewers.docx [file pone.0270145.s003.docx]

Dear reviewer, Dear editorial board member,

We would like to thank you for the positive feedback, and for giving us the chance to revise our work to improve its understandability and readability. We took all the comments into account and made the appropriate changes in the text with the ‘track changes’ mode. The details of the reviewer comments are available below.

Reviewer #1

(Remarks to the Author):

This is an excellent study of an important topic. I have some suggestions to improve clarity outlined below.

METHOD
• I recommend you change the description of the study design (line 13, 81). Currently it reads: ’prospective cross-sectional study with matched controls.’ These are three different designs: prospective implies that it would be a longitudinal cohort study, cross-sectional that it reflects one time point for each individual (cannot be prospective or retrospective), and finally, matched controls that it would be some sort of a case control study. In the statistical section (line 185-6) you refer to the used of the STROBE checklist for case control studies. So, I suggest you choose one of them rather than all three.

Thank you for this recommendation. For more clarity we adapted the text, and the study design is now expressed as “cross-sectional study”.

• According to the method section (line 86-9) children with CP were matched to controls based on age: ’…as well as age matched children with TD. The sample consisted of two groups, children with CP (CP group) and age-matched children with TD (TD group). Children were age matched with peers, with a range of ±1.5 years.’ However, in the discussion, limitations (line 296-7) you state they were matched by age and sex: ’We reduced the induced gait and cognitive differences by matching the patients per age and sex.’ Please revise for consistency.

We would like to thank the reviewer for this comment. We revised the text, as: “*The study population consisted of children with CP between the ages of 8 and 16 years with level I or II according to the Gross Motor Function Classification System (GMFCS) (23), as well as age and sex-matched children with TD.*

*The sample consisted of two groups, children with CP (CP group) and age and sex-matched children with TD (TD group). Children were age matched with peers, with a range of ±1.5 years.*”

• Participants. Dysarthria with difficulties coordinating the muscles used for speaking and coordination of breathing, is more frequent in children with CP. This could potentially influence the tests (cognitive tasks requiring talk: numbers, fruits, animals and be worse while walking rapidly). If none of the children had dysarthria this might be worth mentioning in the method section. If not, it could be mentioned as a limitation.

Thank you for this remark. This is a very important topic. Probably because we merely had children with low GMFCS level, we didn’t have patients with dysarthria. We added it to the method section as you suggested. *“None of the children had dysarthria*.”

RESULTS
• Table 1. According to the characteristics of the participants, the height and especially the mean weight of the study group (CP) exceeds the values of the control group (TD) by 10 kg. This is quite unusual, and typically we see the reversed pattern with more underweight in children with CP than in TP children. Currently the weight range is reported as 21 to 107 kg in the children with CP and that is unusually high. Are these values correct ? If so, please highlight also in the discussion.

We thank the reviewer for this question. These values are correct. As this seems unusual, we had to have a look at the statistical differences between the groups. This was executed with a t-test. It occurs that the two groups were not statistically different. Therefore it seemed important to mention it in the results as: “*The two groups had similar weight (p=0,09) and height (p=0,53*).”

As suggested, we mentioned it in the discussion: “*Also, probably because of the low GMFCS level of the participants with CP, the two groups were statistically similar in weight and height*.”

• Table 3, it is interesting that children with CP seem to have the same cadence as TD children during all three conditions, even though step length is slightly shorter. I think this could be mentioned in the discussion.

Thank you for this suggestion. We took this comment into great consideration and adapted the discussion as: *“*We expected certain differences in gait parameters between both groups. For all three tasks the step length was consistently lower in the CP group. Moreover, the difference between both groups became greater under increased dual-task difficulty. However, we observed that both groups had similar cadence, that decreased similarly in each group when the task became more difficult. It is therefore possible that in children with CP the spatial components of movement programming are more strongly impacted by dual-tasks than its temporal components, when compared to TD children.”

• Figures. Page 6 (line 202) you refer to Figure 1A and 2B for gait stability AP and ML directions. I think this should be Figure 2A and 2B. Figure 1 illustrating Foot Placement Estimator Values is presented at page 4.

*We would like to thank the reviewer for this comment. We could not find this mistake, but we still adapted on the text from « Fig 2 » to « Fig 2A and 2B show the distribution of the two…” for more clarity.*

Other small changes had to be done due to new spotted mistakes or adjustments in order to meet PLOS ONE’style requirement. These are visible in the marked up copy.

We would like to change our Financial Disclosure and Funding Information as: This work was supported by a convention (2015/1) by La Fondation Paralysie Cerebrale (Paris, France, <https://www.fondationparalysiecerebrale.org/>). Sjoerd M.Bruijn was funded by a VIDI grant (016.Vidi.178.014) from the Dutch Organization for Scientific Research (NOW, <https://www.nwo.nl/en>). The funders had no role in study design, data collection and analysis, decision to publish, or preparation of the manuscript."

We would like to adjust the amended statment as : « *The authors thank the participants and their families. We also thank Nathalie Valenza for her contributions in choosing the cognitive tasks and participating in the protocol design. We thank Antoine Poncet for his help on the statistical analysis.”*

We will join the data as supporting information files in the form of two excel documents.

One of the mistakes was about the reference n°39, which is “*Chakraborty S, Nandy A, Kesar TM. Gait deficits and dynamic stability in children and adolescents with cerebral palsy: a systematic review and meta-analysis. Clinical biomechanics. 2019. ». We cited wrongfully « Chakravarthy et all ».*

Best regards,

Sophie Wist
